# Percutaneous Mechanical Circulatory Support Devices in Cardiogenic Shock: A Narrative Review in Light of Recent Evidence

**DOI:** 10.3390/jcm14217731

**Published:** 2025-10-30

**Authors:** Vincenzo Paragliola, Marco Gamardella, Luca Franchin, Maurizio Bertaina, Francesco Colombo, Paola Zanini, Salvatore Colangelo, Pierluigi Sbarra, Giacomo Boccuzzi, Mario Iannaccone

**Affiliations:** 1 Division of Cardiology, School of Medicine and Surgery, Università degli Studi di Tor Vergata, 00133 Rome, Italy; 2 San Giovanni Bosco Hub Emergency Hospital, ASL Città di Torino, 10154 Turin, Italy; 3 Division of Cardiology, School of Medicine and Surgery, Università Vita-Salute San Raffaele, 20132 Milan, Italy

**Keywords:** mechanical circulatory support, cardiogenic shock, myocardial infarction, microaxial flow pump, Impella, VA-ECMO, VA-ECLS

## Abstract

Cardiogenic shock (CS) is a complex, life-threatening syndrome characterized by inadequate tissue perfusion due to impaired cardiac function. Acute myocardial infarction (AMI) and acute decompensated heart failure are the leading causes, with mortality remaining high despite advances in revascularization and supportive care. The Society for Cardiovascular Angiography and Interventions (SCAI) classification allows risk stratification and guides clinical decision making by capturing the spectrum of shock severity. Percutaneous mechanical circulatory support (pMCS) devices, such as the intra-aortic balloon pump (IABP) and Impella, aim to stabilize hemodynamics by augmenting cardiac output and unloading the left ventricle. However, randomized trials and meta-analyses have not demonstrated a consistent survival advantage of Impella over IABP, while reporting higher rates of bleeding and vascular complications. Landmark trials, including ECLS-SHOCK and DanGer, have provided conflicting results, likely reflecting differences in baseline severity and timing of device implantation. Veno-arterial extracorporeal membrane oxygenator (VA-ECMO) offers full cardiopulmonary support but increases left ventricular afterload, potentially worsening myocardial injury. Combined strategies such as ECPELLA (Impella + VA-ECMO) or ECMO + IABP may mitigate left ventricle (LV) overload and improve bridging to recovery or advanced therapies, although evidence remains largely observational and complication rates are considerable. In right-sided or biventricular failure, tailored options (e.g., Impella RP, Bi-Pella) guided by invasive hemodynamics may be required. Current evidence suggests that pMCS benefits are limited to carefully selected subgroups, underscoring the importance of early diagnosis, prompt referral, and individualized intervention. Robust randomized data are still needed to define the optimal role of pMCS in AMI-related CS.

## 1. Introduction

Cardiogenic shock (CS) is a life-threatening syndrome of persistent tissue hypoperfusion due to cardiac pump failure and remains the leading cause of in-hospital mortality after acute myocardial infarction (AMI), as well as a major cause of death in advanced heart failure (HF) [1].

Multiple definitions and diagnostic criteria have been proposed (Table 1). According to the Shock Academic Research Consortium (SHARC) [2], CS is defined as a cardiac disorder resulting in sustained tissue hypoperfusion as identifiable through clinical signs and biochemical markers, irrespective of underlying blood pressure.

Approximately 95% of patients with CS present with severe hypotension requiring intravenous administration of vasopressors and inotropes. However, a subset may maintain normotension despite ongoing tissue hypoperfusion.

Additionally, clinical modifiers such as the mode of presentation (de novo vs. acute-on-chronic) and the extent of myocardial dysfunction (left, right, or biventricular) further contribute to the heterogeneity of this patient population and influence both management strategies and therapeutic outcomes [2,3,4].

The two most frequent causes of CS are acute decompensated heart failure (ADHF) and acute coronary syndrome (ACS), which are responsible for approximately 42% of cases [5,6] and can develop in about 7% of patients referred to the emergency with AMI [7,8], either at presentation or within 24 h thereafter [9]. Despite years of advancement in therapeutic management and the availability of new technologies, morbidity and mortality remain high and unchanged, ranging from 36.3% [10] to 40–50% [11,12]. Given the variability in clinical presentation, experts have developed the Society for Cardiovascular Angiography and Interventions (SCAI) classification scale (Stages A–E) to encompass the full spectrum of cardiogenic shock and standardize risk stratification for both clinical and research purposes. This scale facilitates the assessment of individual risk profiles, comorbidities, in-hospital mortality, response to medical therapy, and suitability for mechanical circulatory support. In addition, serial staging using the SCAI classification serves as a valuable tool for monitoring patient progression over time [13].

Percutaneous mechanical circulatory support (pMCS) refers to device-based therapies designed to mitigate the adverse hemodynamic consequences of myocardial dysfunction, with the goal of progressively restoring adequate tissue perfusion. These devices are engineered for percutaneous implantation, including in emergency settings, and provide temporary or intermediate support to the left and/or right ventricle through automated control algorithms.

The primary function of pMCS is to supplement cardiac output by bridging the gap between actual and required perfusion levels. Hemodynamic stabilization achieved through pMCS results in increased cardiac output, improved coronary and systemic organ perfusion, and reduction of tissue hypoperfusion. Additionally, devices such as the microaxial flow pump (e.g., Impella, Abiomed, Danvers, MA, USA) are capable of unloading the left ventricle, thereby lowering intracavitary filling pressures, reducing myocardial wall stress, and decreasing oxygen demand.

According to European guidelines, mechanical circulatory support should be considered in patients with cardiogenic shock (Class IIa recommendation, Level of Evidence C) [14]; in patients with ACS and severe/refractory CS, short-term mechanical circulatory support may be considered (class IIb recommendation, level of evidence C) [15]. Eventually, mechanical circulatory support may be considered prior to revascularization (class IIb recommendation, level of evidence C) and should be applied before the onset of severe organ dysfunction (class I recommendation, level of evidence B) [1].

**Figure 1:**
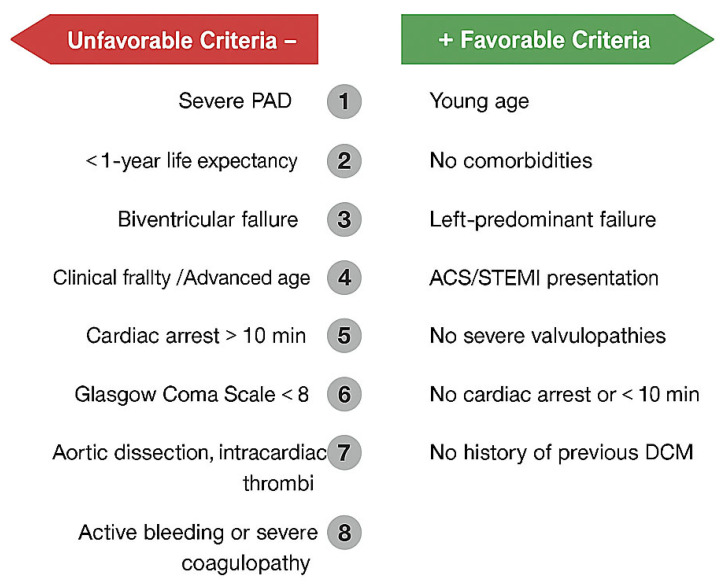
**Factors to be considered for patient selection for pMCS placement.** pMCS: percutaneous Mechanical Circulatory Support, ACS: Acute Coronary Syndrome, STEMI: ST-elevation Myocardial Infarction, DCM: Dilated Cardiomyopathy, PAD: Peripheral Artery Disease.

However, CS survival is frequently determined by extracardiac damage such as anoxic brain damage, multiorgan dysfunction syndrome (MODS) and systemic inflammatory response syndrome [16], and progression to mixed septic shock [17,18]; for these reasons, despite pMCS playing an important role, the optimal timing of initiation, the choice of the proper device in the correct patient is crucial (Figure 1).

For the preparation of this narrative review, a non-systematic literature search was carried out using major biomedical databases, including PubMed, Scopus, and Google Scholar. The search strategy involved the use of medical subject headings (MeSH) and relevant keywords in English, combined using Boolean operators (AND, OR). The selection focused on articles published within the last ten years, prioritizing high-quality sources such as randomized clinical trials (RCTs), systematic reviews, meta-analyses, and evidence-based clinical guidelines. Studies were included based on their clinical relevance, methodological rigor, and contribution to the current understanding of the topic. Preference was given to publications in peer-reviewed journals with a high impact factor. Articles with low methodological quality or lacking relevance to the review objectives were excluded. The final selection aimed to provide a comprehensive and clinically meaningful overview of the available evidence.

## 2. Intra-Aortic Balloon Pump

Intra-aortic balloon pump (IABP) has been used in CS since 1968 [19] and has widely spread during the years, becoming a familiar tool for cardiologists. This device is introduced via femoral arterial access and positioned in the descending aorta. Once activated, it operates in synchrony with the cardiac cycle through alternating inflation and deflation. During systole, deflation reduces left ventricular afterload and wall tension, resulting in a decrease in end-systolic pressure. In diastole, inflation augments diastolic pressure, enhancing blood flow to the coronary arteries and systemic circulation [20,21]. Secondary accesses are the axillary or subclavian artery, either by percutaneous puncture or surgical cutdown [22]. The IABP is estimated to increase cardiac output by approximately 0.5 to 1.0 L/min. However, its efficacy is limited in cases of severely impaired left ventricular function, as it does not provide active myocardial contraction and cannot substitute for intrinsic contractility. Contraindications include severe aortic regurgitation and advanced peripheral vascular disease. The most common complications are related to femoral arterial access, including limb ischemia, cannula malposition, and, in severe cases, embolic stroke.

Early clinical studies indicated a potential benefit of IABP in the acute phase of AMI-CS, particularly when used in conjunction with thrombolytic therapy, by improving coronary perfusion and enhancing fibrinolytic efficacy [23]. The most pivotal randomized controlled trial evaluating the use of IABP in the context of AMI-CS was the IABP-SHOCK II trial. This multicenter study enrolled 600 patients with AMI-CS, all of whom underwent early revascularization—either percutaneous coronary intervention (PCI) or coronary artery bypass grafting—along with optimal medical therapy. Participants were randomized to receive either IABP support (n = 301) or no counterpulsation (control group).

In the IABP group, device implantation occurred either immediately before or shortly after revascularization. The primary endpoint was 30-day all-cause mortality. The results demonstrated no significant difference in mortality between the two groups (39.7% in the IABP group vs. 41.3% in the control group, *p* value = 0.69). This lack of statistical significance persisted across all predefined subgroups, as well as for secondary endpoints, process-of-care measures, and safety outcomes [24]. The 1- and 6-year follow-up data confirmed that IABP placement did not have a significant impact on the survival of AMI-CS patients enrolled [25]. Since the publication of the trial, routine use of IABP in CS has been downgraded as class III in US and European guidelines, maintaining a IIa recommendation (level of evidence C) in case of mechanical complication of AMI [15,26,27].

On the other hand, there is a strong physio-pathological rationale supporting the use of IABP in the context of heart failure-related cardiogenic shock (HF-CS). Ventricular-arteriolar decoupling is a hallmark of this patient population, and IABP appears to mitigate this dysfunction, potentially leading to a greater cardiac output improvement in HF-CS when compared to the AMI-CS one [28]. Some observational studies and small randomized controlled trials seem to support this idea, but a recent randomized controlled trial (RCT) failed to show any benefit [29,30].

The ALT SHOCK 2 trial focused on patients with advanced heart failure admitted for cardiogenic shock, classified as SCAI stages B, C, or D. Participants were randomized to either immediate IABP implantation or standard care with vasoactive therapy and the option for deferred therapeutic escalation. The authors implemented a strict predefined protocol to promptly identify clinical deterioration during hospitalization that would necessitate treatment escalation. The primary outcome was 60-day survival and successful bridging to heart replacement therapies. The trial was suspended at the interim analysis for futility. In addition, there were high rates of bleeding (17%) and vascular complications (7.5%) in the treatment cohort (IABP + standard of care). Several limitations have to be taken into account when interpreting these results. Firstly, the overall survival rate was very high compared to the previous report. This may suggest the enrollment of a low-risk population as demonstrated by a median lactate level below 2 mmol/l, as well as around one-third of patients in the SCAI B stage at admission and a very low inotropic score. Moreover, a non-negligible crossover rate was registered in the pharmacological group. All these factors further limit the trial’s ability to detect differences between treatment arms. However, no signal of benefit emerged, even in secondary or surrogate outcomes. The unique features of HF-CS may warrant a dedicated definition and severity staging before future studies on therapeutic intervention are undertaken. Moreover, a strategy-based, instead of a device-centered RCT, may be more appropriate in the future, considering the confounders and competing risks that play a role, particularly in this group of CS patients [31].

## 3. Microaxial Flow Pump

Microaxial flow pump devices, such as the Impella, are non-pulsatile, axial-flow pumps designed for percutaneous insertion via femoral or axillary arterial access. The device is advanced retrogradely across the aortic valve and positioned within the left ventricle. The Impella CP, for example, is inserted percutaneously using a 14 Fr introducer and can provide up to 4.3 L/min of forward flow. In contrast, the Impella 5.5 is surgically implanted and is capable of delivering up to 5.5 L/min.

The pump functions by drawing blood from the left ventricle and expelling it into the ascending aorta, thereby providing the left ventricle volume–pressure curve and promoting its unloading. The hemodynamic benefits include increased mean arterial pressure, reduced left ventricular end-diastolic pressure and pulmonary artery pressures, and decreased myocardial oxygen demand, all of which contribute to improved cardiac output and systemic perfusion.

Relative contraindications include severe aortic regurgitation, ventricular septal defect, hypertrophic cardiomyopathy, and severe peripheral arterial disease. Absolute contraindications encompass the presence of a mechanical aortic valve prosthesis, mobile left ventricular thrombi or ruptured papillary muscles, and significant calcification or dissection of the ascending aorta [32]. In patients with prohibitive peripheral arterial disease, transcaval access for Impella implantation has been described as a feasible and safe alternative in high-volume centers (DOI: 10.1016/j.jscai.2025.103789)

The Impella CP is increasingly adopted in the management of acute myocardial infarction-related cardiogenic shock (AMI-CS) for short-term mechanical support, as well as in high-risk percutaneous coronary interventions (PCI) [33], while the Impella 5.5 is validated for refractory cardiogenic shock as mid-term support. To date, no randomized controlled trial has evaluated the use of the Impella 5.5, and available evidence is limited to observational studies and registry data [34]. Initial data about Impella 2.5 and CP showed encouraging 30-day survival and safety profile compared to other MCS devices, enhancing its contribution in advanced shock protocols [35,36]. In addition, placement of Impella prior to pPCI emerges as an important feature associated with improved survival in critical patients with extended door-to-balloon time [37]. This favorable trend has been confirmed by a recent meta-analysis in patients receiving Impella CP or 5.0 prior revascularization and without cardiac arrest [38]. In 2024, the results of the DAN-SHOCK (DANger SHOCK) trial were published—an international, multicenter, prospective, randomized, open-label study evaluating the efficacy of microaxial flow pump support in patients presenting with ST-elevation myocardial infarction (STEMI) complicated by cardiogenic shock (CS). A total of 355 patients were enrolled and randomized to receive either standard medical therapy alone (n = 176) or standard therapy plus Impella support (n = 179).

The primary endpoint, all-cause mortality at 180 days post-randomization, was significantly lower in the Impella group (45.8%) compared to the control group (58.5%; *p* = 0.04). This was the first randomized trial in the STEMI-CS population to demonstrate a statistically significant reduction in mid-term mortality with the use of a percutaneous mechanical circulatory support (pMCS), specifically the Impella CP [39]. Common concerns regarding this kind of device are its safety and the frequency of device-related complications. In fact, secondary results from the DanGer trial highlighted higher percentages of safety-composite end point in the mAFP group (24% of total vs. 6.2% in the control group), with a number needed to harm of 6. The list includes major vascular complications like bleeding or limb ischemia, hemolysis, renal-replacement therapy (likely related to hemolysis), and sepsis. Major vascular complications are also a major concern in the previously cited meta-analysis and a common finding in a lot of studies regarding mAFB [40,41]. Yet BARC 3–5 bleedings were not associated with an increase in mortality risk, even when accounting for admission lactate, blood pressure, and LVEF as well as randomization group (HR 0.96; 95% CI 0.67–1.39) as presented in a subanalysis presented at the TCT conference 2024. Moreover, AKI represents a common complication in CS, consequent to hypoperfusion, venous congestion, or sepsis, and a predictor of mortality [42]. In response to these findings, the authors conducted a secondary analysis focusing on acute kidney injury (AKI), the use of renal replacement therapy, and their associations with mortality. AKI occurred in 189 patients (53% of the total cohort, including 61% of those allocated to pMCS and 45% of those in the control group; *p* < 0.01) and was linked to poorer baseline clinical status and a worse prognosis. Treatment with the Impella device was associated with an increased incidence of both AKI and RRT, primarily attributed to suction events and the provision of more prolonged and intensive support. Despite these adverse renal effects, the 180-day survival benefit observed in the pMCS group outweighed the negative impact of AKI and RRT. The authors therefore suggested that a better understanding of the mechanisms predisposing patients to device-related AKI and RRT may inform clinical strategies to mitigate these risks and ultimately improve overall outcomes [43]. Apart from the significant exceptional findings in this field, it is mandatory to list some limiting considerations. Inclusion criteria were very strict and excluded most of AMI-CS presenting to enrollement centers (nearly 70% of screened patients). In fact, the enrollement was very slow and took more than 10 years to complete with the contributions of more centers. On the one hand, results can apply only to a limited category of acute patients, limiting also the comparison with other trials. On the other hand, heterogeneity and treatment bias could have influenced the results, even considering the unblinded nature of the trial. Absence of a definite treatment protocol together with discharge criteria affects the reproducibility of clinical outcomes, some of which influence primary end-points. Hence, higher rates of clinical complications associated with mAFP are likely influential to different ICU treatment strategies and intensity displayed among the two groups.

One aspect not addressed in the DanGer Shock trial is the timing of device implantation. In recent years, the concept of door-to-unloading has emerged, referring to the placement of the Impella device in patients with AMI-CS prior to performing primary coronary revascularization. This strategy aims to firstly unload the left ventricle during the period of coronary occlusion. Although this approach inherently prolongs ischemic time, preliminary evidence from registries and meta-analyses suggests a potential benefit in terms of reducing infarct size—primarily through a decrease in myocardial oxygen demand—and mitigating the progression of the shock cascade compared to the conventional revascularization-first strategy [44,45]. Moreover, the effect of Impella CP appeared to be greater in patients with less advanced shock (SCAI stage C), lower mean arterial pressure (≤63 mmHg), and multivessel coronary artery disease, although these subgroup analyses were not powered for definitive conclusions [38]. Indeed, in the DanGer Shock trial, subanalysis showed that microaxial flow pump placement was correlated to a faster lactate clearance [46]. Basir et al. assessed the safety and feasibility of this practice with a large single-arm multicenter study enrolling CS patients (SCAI ≥ C) addressed to pMCS within a definite therapeutic shock protocol; moreover, survival rates turned out to be encouraging compared to previous ones seen in other trials [47]. Contemporary meta-analysis focused on the timing of Impella placement in this setting, regarding studies with both Impella CP and Impella 2.5, revealed a significant reduction in short-term and mid-term mortality in patients receiving Impella pre-PCI, without increasing the complication rate [48]. Long-term follow-up up to 10 years confirmed the survival benefit of Impella CP, with an absolute mortality reduction of 16.3% and a mean gain of approximately 600 days of life [49].

Data from the ongoing STEMI-DTU trial could become a turning point in some specific settings of AMI [50].

On the other hand, right ventricular (RV) failure is often an under-recognized issue in the setting of cardiogenic shock and is associated with increased in-hospital mortality. However, previous data have been conflicting, depending on the underlying etiology of shock and the hemodynamic criteria used to define right ventricular (RV) failure. RV dysfunction typically occurs alongside left ventricular impairment, while isolated RV failure is less commonly observed [51,52].

Peculiar signs and symptoms are jugular vein distension, paradoxical pulse, hepatomegaly, and peripheral congestion. Echocardiography may offer valuable clues for identifying it, but invasive hemodynamic assessment with a Swan-Ganz catheter is often more appropriate for a definitive diagnosis [53,54,55]. It is worth noting that Impella technology is available for a specific RV support device named Impella RP (Abiomed, Danvers, MA, USA). The system exploits a 22 French impeller and an 11 French catheter inserted through a 23 French peel-away introducer and is built to place the inflow part in the inferior vena cava and the outflow part in the pulmonary artery, so that it bypasses the RV itself. The newer version, named Impellla RP “Flex”, demands an insertion through the internal jugular vein (still not available in Europe) [56]. The RECOVER RIGHT trial demonstrated the safety and feasibility of this device implantation in patients with hemodynamic criteria of RV failure, highlighting the hemodynamic benefit during hospitalization in 30 selected patients [57]. Despite this initial supporting evidence, limited experience is available until now, particularly when this device is combined with LV ones in the biPella configuration in case of biventricular failure. Moreover, the impact of LV support by itself on the RV dysfunction has still to be clarified [58].

## 4. Tandem Heart

The TandemHeart system (LivaNova, London, UK) is a percutaneous circulatory support device consisting of an external centrifugal pump and two cannulas, designed to provide continuous blood flow by delivering oxygenated blood from the left atrium to the arterial system via the femoral artery. The inflow cannula is introduced into the left atrium through transseptal puncture, typically requiring a 21F introducer sheath. A 15F (maximal estimated flow of 3.5 L/min) or 19F (maximal estimated flow of 4.0–5.0 L/min) outflow cannula is then advanced to the iliac artery by percutaneous femoral arterial access. The system is capable of delivering up to 5.0 L/min of flow, thereby reducing left ventricular (LV) pressure and volume, decreasing myocardial oxygen consumption, and enhancing peripheral perfusion in a sustained manner. Clinical data on TandemHeart use remain limited, primarily derived from isolated case series in selected centers. However, an observational study involving 50 patients with cardiogenic shock undergoing TandemHeart implantation demonstrated early hemodynamic improvement and promising survival outcomes, including in patients ultimately undergoing surgical intervention. These findings support the safety and feasibility of the device and provide a rationale for future, more rigorous clinical investigations [59]. Two studies compared the hemodynamic effect of this device to IABP, finding better hemodynamic support (higher cardiac power index and reduced pulmonary wedge pressure) but together with increased rates of complications [60,61].

## 5. VA-ECMO

Veno-arterial extracorporeal membrane oxygenator (VA-ECMO) is a percutaneous mechanical circulatory support device that provides both full circulatory and respiratory support. It consists of a closed circuit incorporating a membrane oxygenator and a centrifugal pump, which drains deoxygenated blood from the venous system and returns oxygenated blood to the arterial circulation. Vascular access is achieved through the insertion of a large-bore venous cannula (17–24 Fr) and an arterial cannula (14–21 Fr) [62]. VA-ECMO offers the most comprehensive level of support among temporary devices, delivering assistance with flow rates of up to 6 L/min alongside concurrent blood oxygenation [63].

The underlying mechanism of VA-ECMO differs from other support systems, as it effectively bypasses the heart, thereby providing systemic perfusion independently of native cardiac output. However, this configuration inevitably impacts native hemodynamics. Although venous return is reduced, VA-ECMO flow increases left ventricular (LV) afterload and end-diastolic pressure. In the context of severely impaired myocardial function, native stroke volume is often markedly diminished, raising the risk of LV or aortic thrombus formation due to stasis. The addition of a second device, such as an intra-aortic balloon pump (IABP) or Impella, may alleviate these adverse effects by facilitating LV unloading [64]. The recently described LAVA-ECMO strategy, as reported by Giustino et al., provides an alternative approach to reduce LV afterload, thereby achieving effective unloading and mitigating the hemodynamic limitations of conventional VA-ECMO [65].

VA-ECMO has also been investigated as an adjunctive therapy during cardiopulmonary resuscitation (ECPR), and in this context, has received a Class II, Level C recommendation in the ESC guidelines for acute coronary syndromes [15]. Contraindications to VA-ECMO include severe peripheral arterial disease and significant aortic regurgitation. Common complications include bleeding, limb ischemia, pump thrombosis, Harlequin syndrome (differential hypoxemia), pulmonary edema, and sepsis [66].

European Society of Cardiology regulates VA-ECMO in a position statement recommending the use of this device in selected patients with refractory cardiogenic shock caused by acute myocardial infarction [67], overwhelming the small scientific evidence in this setting. Recently, the EURO-SHOCK trial aimed to compare VA-ECMO with standard care following primary PCI; however, recruitment was significantly impacted by the COVID-19 pandemic, and the limited number of enrolled patients compromised the reliability of the conclusions. However, preliminary data showed numerically lower rates of 30-day and 1-year mortality for patients treated with VA-ECMO [68]. Similarly, the ECMO-CS trial randomized 117 patients presenting with SCAI stage D or D–E cardiogenic shock to immediate initiation of VA-ECMO or to a conservative strategy comprising non-invasive and pharmacological therapy. The study population was heterogeneous, with acute myocardial infarction representing the most common etiology. At 30-day follow-up, there was no statistically significant difference between the two groups in the primary composite endpoint—comprising all-cause mortality, resuscitated circulatory arrest, and escalation to another mechanical circulatory support device—or in all-cause mortality alone (50.0% in the VA-ECMO group vs. 47.5% in the conservative group). These findings were consistent across all etiological subgroups.

Notably, the safety profile demonstrated comparable rates of serious adverse events in both arms. The interpretation of outcomes is complicated by the considerable rate of bailout VA-ECMO use among patients in the conservative treatment arm experiencing clinical deterioration. Nevertheless, the findings align with existing evidence regarding the clinical trajectory and management of patients with severe cardiogenic shock [69]. More robust evidence is provided by the largest ECLS-SHOCK trial, which randomized 420 patients with AMI-related cardiogenic shock to receive either VA-ECMO in addition to standard therapy or standard therapy alone. The trial did not demonstrate a statistically significant difference in the primary endpoint of all-cause mortality at 30 days (47.8% in the VA-ECMO group vs. 49.0% in the control group), nor in the key secondary clinical endpoints. Conversely, the use of VA-ECMO was associated with a higher incidence of complications, particularly vascular events.

The trial population included a substantial proportion of patients in advanced stages of shock, with three-quarters having undergone cardiopulmonary resuscitation and most likely sustaining cerebral injury or detrimental multi-organ irreversible damage. From the therapeutic management point of view, there was a non-negligible crossover rate between the two groups, since 12.5% of control patients underwent VA-ECMO insertion due to worsening conditions, most of them within 24 h after randomization, whereas 8.1% of patients randomized to ECMO did not receive the therapy. Conversely, not considering a device-oriented strategy but an MCS-strategy, it appears influential the significant rate of other forms of tMCS in the control group (15.6%), adds to the previous crossover quote. Plus, implementation with LV unloading in the ECMO group was very low (5.8%), especially compared to the rates displayed by other similar studies. These factors have been advocated as potential confounders that may have attenuated any potential survival benefit of VA-ECMO in this setting [70]. The absence of clinical benefit at 30 days was further corroborated by the meta-analysis conducted by Zeymer et al., which included all four available randomized controlled trials. Among the 565 patients analyzed, 46% (n = 129) in the VA-ECMO group and 48% (n = 135) in the control group died within 30 days. Approximately two-thirds of the study population (68%) presented with ST-elevation acute coronary syndrome, and baseline characteristics reflected a very advanced and compromised cohort of CS patients: 67% had undergone cardiopulmonary resuscitation prior to randomization, 63% had multivessel coronary artery disease, and a considerable proportion experienced crossover or escalation to an additional mechanical circulatory support device.

Notably, only a minority of patients received VA-ECMO prior to the index percutaneous coronary intervention, and no statistically significant differences were identified with respect to the timing of device implantation. From the safety point of view, the harmful effect of VA-ECMO has been questioned, considering the observed increase in bleeding (BARC 3–5), peripheral vascular complications, ventilation time, and inflammatory response [70]. Even if it was not always associated with prolonged intensive care unit treatment and total in-hospital length of stay, this kind of complications represents an important concern, as they worsen survival. These findings highlight the uncertainty regarding the potential benefit of VA-ECMO in such critically ill patients, where the line between hope of treatment benefit and futility appears to be very thin [71].

## 6. Comparative Analysis

Scientific evidence on different PMCS covers several years of practice, and some data regards comparative use of different devices in the same setting of CS (mainly AMI-CS). For years, IABP has been the most widely used mechanical support in shock, and with the advent of Impella and its different rationale, some trials have compared their mutual efficacy and safety (Table 2). Although Impella proved superior hemodynamic support compared to IABP with higher CI, higher mean arterial pressure, and lower pulmonary capillary wedge pressure immediately after implantation, related to a net metabolic gain, initial data did not demonstrate a translation to significant mortality rate reduction at 30 days [72]. That was confirmed by a subsequent meta-analysis taking into account small trials comparing pLVAD (Impella 2.5 and TandemHeart) to IABP [73]. The major limitations of these studies must be mentioned: the results are extracted from small samples of patients, showing significant heterogeneity in terms of shock phenotyping, timing of implants, and clinical assessment. Plus, the results might be considered outdated in light of newer devices (Impella CP) and major expertise. The first evidence coming from larger samples of comparative strategies comes from a retrospective analysis performed in patients treated with Impella matched to those enrolled in the IABP-SHOCK II trial, aligning with previous results on early mortality rates (30 days) at the cost of a higher incidence of bleeding and severe vascular complications with the use of Impella. Still, the method’s limitations limit the level of evidence provided, as it tries to compare two unrelated groups created in a different fashion. The matching procedure tries to overcome this limit; however, the result is that the Impella population reflects the same criticisms, by including patients despite recovery potential measures and not considering door-to-support timing [41].

A more recent exploratory randomized trial compared Impella CP to IABP in mechanically ventilated patients with AMI-related cardiogenic shock. The study enrolled a total of 48 patients and found no statistically significant differences in mortality at 30 days or at 6 months between the two treatment arms. Notably, the rate of severe bleeding was higher in the Impella CP group (26% vs. 8%). However, the trial was clearly underpowered to detect meaningful differences in either primary or secondary endpoints. Moreover, the advanced clinical condition of the enrolled population may have masked any potential benefit of mechanical ventricular support [74]. No significant reduction in 30-day mortality or clinical benefit with the use of Impella instead of IABP has emerged from more recent matched comparisons and meta-analysis, besides higher percentages of severe bleeding and vascular complications were registered. Device-related complications in this population can overcome the benefit gained by the circulatory support granted by Impella; thus, a more specific selection of these patients and longer follow-up could highlight different outcomes [40,75,76]. It has to be taken into account that all the comparative data between microaxial flow pump and IABP are mostly with the use of the Impella 2.5 device, which is no longer on the market, and were performed in centers with limited initial experience prior to the Danger shock RCT publication. Plus, Impella hemodynamics’ advantage over IABP is perceptively clear and widely found in previous data, so focusing on early mortality outcomes might be restrictive, as increased hemodynamic performance during the acute phase could exert clinical benefit that emerges in a longer follow-up period.

ECLS-SHOCK and DanGer Shock represent two pivotal trials in the field of mechanical circulatory support, providing extensive data from well-characterized patient populations and anticipated to offer definitive guidance for clinical practice. However, their findings remain conflicting, with notable differences in outcomes. A recent report addressed these discrepancies, highlighting several factors that may explain the divergent results beyond the intrinsic benefits of the respective devices.

Primarily, the patient cohort in ECLS-SHOCK exhibited more severe baseline clinical characteristics—including higher SCAI classification, elevated lactate levels, and a greater incidence of cardiac arrest with poor neurological outcomes following return of spontaneous circulation—which likely contributed to the increased early mortality observed. Additionally, mortality reduction associated with Impella therapy appeared to increase progressively over time, reaching statistical significance at 180 days. This effect becomes even more pronounced at longer follow-up, which may reflect the contribution of hemodynamic stabilization through pMCS to organ preservation [49].

Conversely, mortality rates in the VA-ECMO group remained consistently higher at early follow-up compared to Impella and did not substantially improve over prolonged observation [77]. The authors proposed that these differing temporal mortality patterns may be attributable to the earlier intervention strategy employed in DanGer Shock, where 55% of devices were implanted prior to index PCI, compared to only 21.9% in ECLS-SHOCK. This suggests that the systemic benefits of mechanical circulatory support are optimized when initiated before the development of advanced shock and metabolic derangements. That said, the higher proportion of patients with SCAI stage E cardiogenic shock (32.1% in ECLS-SHOCK vs. 15.6% in DanGer Shock) and those achieving return of spontaneous circulation (ROSC) prior to randomization (77.5% in ECLS-SHOCK vs. 21.8% in DanGer Shock) may have mitigated any potential systemic benefits of mechanical circulatory support (MCS), with delays in initiation likely reflecting more severe baseline conditions rather than differences in timing alone [78]. Moreover, it must be mentioned that the use of VA-ECMO is responsible for increased LV afterload, which sums up to impaired hemodynamics in the acute phase of CS, so that eventual adjunction of unloading devices (IABP or Impella) can compensate for this detrimental effect and ameliorate global status [79,80].

Meaningful comparative evidence regarding percutaneous MCS in AMI-CS derives from an individual patient data meta-analysis performed by Thiele et al. of nine randomized controlled trials, in which VA-ECMO- or Impella-treated patients were analyzed until extended follow-up at 6 months, with the intent to study early routine utilization of mechanical support. No significant reduction in all-cause mortality emerged either with unselected use of MCS and subcategories of isolated unloading or loading device, compared to standard care. In contrast, analysis filtered by patient selection found significant mortality benefit in subgroups at low risk for hypoxic brain injury (44.3% vs. 55.3%), as well as in patients with TIMI flow < 3 post-revascularization (hazard ratio [HR] 0.57) and systolic blood pressure < 80 mmHg (HR 0.63). Consistent with prior evidence, patients receiving mechanical circulatory support experienced higher rates of severe bleeding (odds ratio (OR) 2.64) and ischemic vascular complications (OR 4.43). Interestingly, even MCS initiation timing in relation to PCI did not influence survival rate. According to the authors, these data can be explained by the multifactorial dependence of mortality in an acute setting, in which hypoperfusion is not the only agent. Therefore, higher complications associated with pMCS may outweigh the hemodynamic support benefit. On the other hand, selecting patients at low risk of neurological compromission, simulating a population of DanGer Shock fashion, finds an increased survival rate with the application of MCS. Assuming this feature as a key factor, it is suggested that early MCS in selected refractory cardiogenic shock can effectively improve prognosis. Data explanation passes by clear limitations of the state of the art, which count heterogeneous trial designs—regarding inclusion criteria, treatment protocols, device-oriented analysis—and poor enrollment. Specifically, the data extracted are mainly driven by two major trials in the field (ECLS-Shock and DanGer), with lighter contributions by other small studies, whose differences have previously been highlighted [81].

## 7. Combined Strategy

VA-ECMO is widely utilized as an effective modality of support, particularly in cases of cardiac arrest or refractory cardiogenic shock. However, its application is frequently complicated by adverse hemodynamic effects related to left ventricular (LV) overload. In patients with impaired LV function, the ventricle may fail to accommodate increased preload, resulting in retrograde congestion in the left atrium and pulmonary circulation. This leads to elevated myocardial oxygen demand, which impairs myocardial recovery and contributes to progressive deterioration of ventricular pump function, ultimately resulting in blood stasis.

To mitigate these deleterious effects, the concurrent implementation of LV unloading strategies during VA-ECMO has been proposed to improve clinical outcomes. Among percutaneous mechanical circulatory support devices, Impella represents the most effective LV unloading option and can be used in combination with VA-ECMO (termed ECPELLA). Preliminary studies comparing ECPELLA with VA-ECMO alone have suggested reductions in early mortality rates and increased rates of bridging to myocardial recovery or advanced therapies [82,83]. The protective effect of the ‘ECPELLA’ configuration appears to be time-dependent, with early initiation associated with better outcomes compared to delayed implementation [84]. Positive trend was confirmed by a meta-analysis, observing a reduction in 30-day mortality and a higher rate of recovery; however, the presence of two contemporary MCS devices induces major bleeding events and higher clinical complications possibly [85]. One other possibility would be to combine VA-ECMO, with IABP. This setting should reduce vascular complications and hemolysis driven by Impella, but of course would achieve a lower LV unloading. Interestingly, Low et al. performed a network meta-analysis including 48,749 patients deriving from RCTs and propensity score-matched studies, in which data belonging to different combined MCS strategies were collected (ECMO, ECMO-IABP, ECMO-mVAD, IABP, mVAD, mVAD-IABP, centrifugal VAD (TandemHeart, cVAD), medical therapy alone). Each mechanical circulatory support (MCS) strategy was compared to no MCS therapy, with a significant reduction in early mortality observed only for the combination of ECMO and IABP. This finding gains further relevance considering that only patients classified as SCAI stages C to E—representing the most critically ill subset of cardiogenic shock—were included. The superiority of ECMO combined with IABP over ECMO alone aligns with the physiological rationale of adding ventricular unloading to the life-sustaining circulatory support provided by ECMO.

However, adverse events such as bleeding, limb ischemia, and acute kidney injury (AKI) are variably associated with each MCS modality. Despite including several high-quality studies with large patient populations, the results are limited by heterogeneity in inclusion criteria, the lack of data from randomized controlled trials (RCTs) (with most evidence deriving from propensity score-matched analyses), and the absence of standardized data collection protocols [86].

Most available data on this topic come from observational and retrospective studies, often based on device-specific analyses with limited and heterogeneous case series, and frequently lacking important clinical variables. As with other MCS configurations, the timing of initiation appears to be a key determinant of outcomes and should be standardized in future protocols. Similarly, different SCAI stages, underlying etiologies, myocardial recovery potential, and comorbidities likely require tailored approaches to MCS selection and management.

In clinical practice, the use of dual mechanical support is typically reserved for the most critically ill patients who fail to improve during the acute phase with single-device support. Given the increased cost and higher risk of complications, the second device (usually IABP) is often implemented as an upgrade. To date, no definitive clinical trial has been conducted to determine the optimal patient selection criteria, ideal timing of initiation and escalation, or best practices for acute management of combined MCS strategies.

Therefore, it may be hypothesized that combining ECMO with a ventricular unloading device promotes myocardial recovery and reduces left ventricular remodeling, thereby improving mid- to long-term outcomes. Nonetheless, the complexity of managing simultaneous support systems and the higher incidence of complications raise concerns about the cost-effectiveness of this approach, particularly outside high-volume, high-expertise centers.

## 8. Gaps and Future Directions

The field should pivot from device-centric to strategy-centric trials—randomizing approaches such as pre-PCI unloading vs. immediate revascularization and protocolized shock-team pathways, rather than single devices. Endpoints must extend beyond 30-day mortality to functional/neurologic recovery, quality of life, and days alive out of hospital. Dedicated RCTs of right-ventricular (and biventricular) support are also needed. Ongoing studies (e.g., STEMI-DTU, NCSI-II, ANCHOR examining ECMO + IABP, UNLOAD ECMO examining ECMO + Impella) will provide interesting results, but they should be interpreted cautiously—with attention to design limits and confounding.

This work presents limitations typical of narrative reviews. The lack of a systematic methodology for literature selection may introduce bias and reduce reproducibility. Furthermore, the authors’ quality assessment of included studies may reflect personal biases, potentially weakening the reliability of the conclusions. Therefore, although efforts were made to apply an objective approach, work should be interpreted with critical analysis by expert readers, especially in clinical contexts, using established guidelines as the primary reference.

## 9. Conclusions

Early referral, prompt diagnosis, and timely intervention are critical determinants of survival in patients with AMI-CS. Given the substantial hemodynamic benefits conferred by pMCS, these advantages must be balanced against an increased risk of potentially fatal complications. Consequently, optimal management requires careful identification of clinical scenarios in which pMCS deployment is most likely to confer net benefit. Current debates focus on precise phenotyping and stratification of cardiogenic shock, identification of prognostic modifiers—including biochemical markers, hemodynamic indices, and clinical signs—and determination of the most appropriate timing for device implantation. Device-oriented trials suffer from impactful selection and heterogeneity bias, with the result that positive outcomes may be reproducible only in a limited setting. Instead, management should prioritize phenotype- and timing-guided strategies within a multidisciplinary shock-team framework, with vigilant complication prevention and early reassessment [87]. Considering different specifications of each device, physicians should properly collect this information and assess the clinical and hemodynamic benefits that each device could bring, even resorting to implementing combined strategies.

## Figures and Tables

**Table 1:** **Cardiogenic shock definition.** Legend: ACVC—Association for Acute Cardio Vascular Care; ESC—European Society of Cardiology; LVEDP—left ventricular end diastolic pressure; PCWP—pulmonary capillary wedge pressure; ALT—alanine aminotransferase.

ESC Heart Failure Guidelines 2021	Diagnosis of cardiogenic shock mandates the presence of clinical signs of hypoperfusion (cold extremities, oliguria, mental confusion, dizziness, narrow pulse pressure).	Biochemical manifestations of hypoperfusion (elevated serum creatinine and lactate, metabolic acidosis). Hypoperfusion is not always accompanied by hypotension.
ACVC position statement 2020	All of the following: Systolic blood pressure < 90 mm Hg for >30 min OR need of vasopressors to maintain systolic pressure > 90 mm Hg	Clinical signs/symptoms (at least one of the following): Altered mental status OR cold, clammy skin and extremities OR oliguria with urine output < 30 mL/h OR arterial lactate >2.0 mmol/L And elevated left ventricular filling pressures: Pulmonary congestion OR PCWP/LVEDP > 20 mm Hg
Shock Academic Research Consortium (*Clinical trial def*)	*CLINICAL PRACTICE DEFINITION* Cardiac disorder that results in both clinical and biochemical evidence of sustained tissue hypoperfusion. *CLINICAL TRIAL DEFINITION* Systolic blood pressure < 90 mm Hg for ≥30 min (or the need for vasopressors, inotropes or mechanical circulatory support to maintain systolic blood pressure ≥ 90 mm Hg) with evidence of hypoperfusion.	Hemodynamic criteria * (optional) Cardiac index < 2.2 L/min/m^2^ AND Hypoperfusion (1 of the following): arterial lactate >2 mmol/L, acute kidney injury (creatinine ≥2× upper limit of normal) OR oliguria (e.g., urine output < 0.5 mL/(kg·h), acute hepatic injury (e.g., ALT > 3× upper limit of normal), cool or mottled extremities, altered mental status not explained by an alternative cause.

* Note: Criteria valid even in the absence of hypotension if clinical hypoperfusion is present with evidence of severe ventricular dysfunction.

**Table 2:** **Temporary mechanical support device hemodynamic effect.** Legend: CO—Cardiac Output; CPO—Cardiac power output; CVP—Central venous pressure; LVEDP—Left ventricular end diastolic pressure; MAP—Mean arterial pressure; PCWP—Pulmonary Capillary wedge pressure; IAPB—Intra-aortic balloon pressure; VA-ECMO—Veno-Arterial Extra-Corporeal Membrane Oxygenation.

	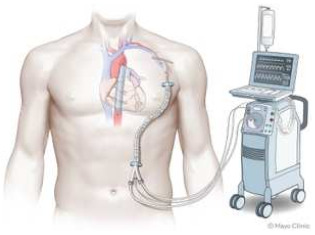	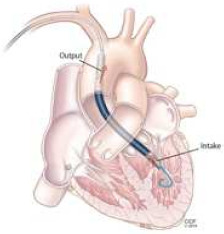	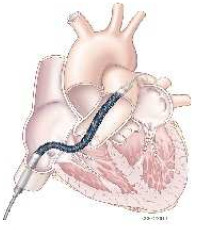	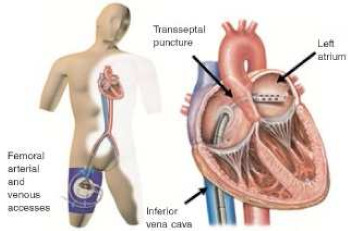	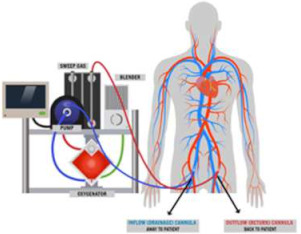
	**IAPB**	**IMPELLA CP**	**IMPELLA RP**	**TANDEM HEART**	**VA-ECMO**
**CO**	↑	↑↑	↑↑	↑↑	↑↑↑
**CVP**	no effect/↓	no effect/↓	↓↓	↓↓	↓↓
**PCWP/LVEDP**	no effect/↓	↓↓	no effect/↑	↓↓	↑
**MAP**	↑	↑↑	↑↑	↑↑	↑↑↑
**TOTAL CPO**	no effect/↑	↑↑	↑↑	↑↑	↑↑↑
**AFTERLOAD**	↓	no effect	no effect	↑↑	↑↑
**SAT (O2)**	no effect	no effect	no effect	no effect	↑↑
**SHEAT SIZE**	Arterial, 7–8 Fr	Arterial, 14 Fr	Venous	Arterial, 15–19 Fr Venous, 21 Fr	Arterial, 15–17 Fr Venous, 21–23 Fr

## Abbreviations

ACS	Acute coronary syndrome
AKI	Acute kidney injury
AMI	Acute myocardial infarction
CS	Cardiogenic shock
HF	Heart failure
IABP	Intra-aortic balloon pump
LV	Left ventricle/left ventricular
MAP	Mean arterial pressure
MCS	Mechanical circulatory support
MODS	Multiorgan dysfunction syndrome
mAFP	Microaxial flow pump
OR	Odds ratio
PAD	Peripheral arterial disease
pMCS	Percutaneous mechanical circulatory support
PCI	Percutaneous coronary intervention
RV	Right ventricle/right ventricular
STEMI	ST-elevation myocardial infarction
VA-ECMO	Veno-arterial extracorporeal membrane oxygenation
